# Machine Learning Applied to Clinical Laboratory Data in Spain for COVID-19 Outcome Prediction: Model Development and Validation

**DOI:** 10.2196/26211

**Published:** 2021-04-14

**Authors:** Juan L Domínguez-Olmedo, Álvaro Gragera-Martínez, Jacinto Mata, Victoria Pachón Álvarez

**Affiliations:** 1 Higher Technical School of Engineering University of Huelva Huelva Spain; 2 Juan Ramón Jiménez University Hospital Huelva Spain

**Keywords:** COVID-19, electronic health record, machine learning, mortality, prediction

## Abstract

**Background:**

The COVID-19 pandemic is probably the greatest health catastrophe of the modern era. Spain’s health care system has been exposed to uncontrollable numbers of patients over a short period, causing the system to collapse. Given that diagnosis is not immediate, and there is no effective treatment for COVID-19, other tools have had to be developed to identify patients at the risk of severe disease complications and thus optimize material and human resources in health care. There are no tools to identify patients who have a worse prognosis than others.

**Objective:**

This study aimed to process a sample of electronic health records of patients with COVID-19 in order to develop a machine learning model to predict the severity of infection and mortality from among clinical laboratory parameters. Early patient classification can help optimize material and human resources, and analysis of the most important features of the model could provide more detailed insights into the disease.

**Methods:**

After an initial performance evaluation based on a comparison with several other well-known methods, the extreme gradient boosting algorithm was selected as the predictive method for this study. In addition, Shapley Additive Explanations was used to analyze the importance of the features of the resulting model.

**Results:**

After data preprocessing, 1823 confirmed patients with COVID-19 and 32 predictor features were selected. On bootstrap validation, the extreme gradient boosting classifier yielded a value of 0.97 (95% CI 0.96-0.98) for the area under the receiver operator characteristic curve, 0.86 (95% CI 0.80-0.91) for the area under the precision-recall curve, 0.94 (95% CI 0.92-0.95) for accuracy, 0.77 (95% CI 0.72-0.83) for the F-score, 0.93 (95% CI 0.89-0.98) for sensitivity, and 0.91 (95% CI 0.86-0.96) for specificity. The 4 most relevant features for model prediction were lactate dehydrogenase activity, C-reactive protein levels, neutrophil counts, and urea levels.

**Conclusions:**

Our predictive model yielded excellent results in the differentiating among patients who died of COVID-19, primarily from among laboratory parameter values. Analysis of the resulting model identified a set of features with the most significant impact on the prediction, thus relating them to a higher risk of mortality.

## Introduction

The COVID-19 pandemic is one of the most prominent health catastrophes of the modern era. This is not exclusive to the health field, as the far-reaching economic and social consequences of this crisis are still unquantifiable [1]. The disease primarily affects the respiratory system, causing respiratory failure, and in certain patients, results in severe inflammatory syndrome. This is mediated by proinflammatory cytokines and can lead to marked systemic complications, which may be fatal in many cases [2].

The lack of knowledge of this virus led the World Health Organization, together with the US Center for Disease Control and Prevention, to define a profile of high-risk patients; this included factors such as age over 65 years, living in nursing homes, and having at least one of the following health problems: chronic lung disease, severe heart disease, obesity, diabetes, kidney failure, liver disease, or an immunocompromised status. The result has been a highly uneven response to the pandemic, both with respect to treatment and the diagnostic and prognostic criteria for the disease [3].

The exponential increase in COVID-19 cases over a short period, lack of experience and knowledge of the virus, and the shortcomings in health resources and health care personnel (many of whom were infected) have caused hospitals to become saturated, especially intensive care units, which have received a very high number of patients every day, many of whom required long stays. Pressure on the health care system after the first wave of the pandemic led to a search for different resources in order to help understand and accurately predict how each patient would react on interacting with the virus. The availability of tools to enable us to classify at-risk patients is crucial because microbiological diagnostics are slow, the PCR test takes more than 4 hours, and emergency physicians do not usually receive the results for up to 24 hours after collecting the sample. Furthermore, the treatments are based on vital support that is not always effective, and potentially gives rise to a large number of adverse events; furthermore, drug availability is sometimes limited. Developing tools that allow us to classify patients at the risk of complications, such those with a prothrombotic status, or an increase in the number of inflammation parameters in a blood sample, would help alleviate the saturation of the health system, optimize resources, and save time in resolving clinical complications [4].

Hence, we developed a model to predict the mortality risk from the laboratory parameters obtained during patients’ hospital stay [5]. With this model, we aimed to evaluate how laboratory parameters are related to the risk of a more (or less) severe disease, so that when a patient presents at the emergency department at a hospital, the mortality risk can be predicted on the basis of the blood parameters.

## Methods

### Data Description

This study is based on anonymized clinical data obtained from a private hospital group in Spain (HM Hospitales), with centers primarily in the Autonomous Communities of Madrid and Galicia and in Barcelona. This group made its data available to the scientific community for research purposes. Using these electronic case histories, we accessed data on individuals suspected with COVID-19 admitted to their centers between March and June 2020. From all the data tables provided, we selected the following: (1) a main table containing specific data on hospitalization and patients (2547 records) and (2) a laboratory data table with the results of the various tests requested for each patient during hospitalization and those presenting at the emergency department (584,136 records).

In the table, an “Outcome” feature is present, with 5 possible values: “Death,” “Home,” “Transfer to hospital,” “Transfer to sociosanitary center” and “Voluntary discharge.” This Outcome feature is the aim of the predictive model developed in this study.

### Data Preprocessing

Before developing the model, and as a prior step in any machine learning procedure, the information in the 2 tables was preprocessed as follows:

1. Only those patients with a confirmed diagnosis of COVID-19, and whose “Outcome” feature was either “Home” or “Death,” were selected.
2. Data from both tables were combined in accordance with the patient ID. Since the patients can present a variable number of measurements for each laboratory parameter, the mean value was calculated and assigned to each of them.
3. Owing to the large number of missing values, we decided to filter records and features in order to handle data without missing values. Some machine learning algorithms can function by directly using data with missing values, and imputation methods can also be used. In this study, for the sake of uniformity and simplicity, the following procedure was used: first, those features having missing values in >10% of all records were eliminated; thereafter, only those records that had value in all the remaining features were selected.
4. Features such as “Sex” and “Outcome” were properly encoded as binary values. No other preprocessing such as normalization or scalarization was applied to the data.

### Machine Learning Techniques

A range of machine learning methods to obtain predictive models have been developed, such as those based on logistic regression, linear discriminant analysis, instance-based learning, artificial neural networks, decision trees, and ensemble learning. This study applied the gradient boosting method to develop a predictive model.

#### Gradient Boosting

Gradient boosting is a machine learning technique used to resolve regression and classification problems and yields a predictive model through an ensemble of weak prediction models, usually decision trees. As in other boosting methods, it builds the model incrementally by incorporating weak prediction models, but it optimizes an arbitrary differentiable loss function. Finally, the prediction for a new case is obtained by aggregating the predictions of all the individual decision trees that constitute the model. By combining many trees, nonlinearity and interactions between predictor features are achieved [6].

Extreme gradient boosting (XGBoost) is a relatively new gradient boosting implementation that has achieved excellent results in many classification tasks. It is an open-source software library that provides a gradient boosting framework designed to be highly efficient and flexible [7]. It has also been successfully applied in medicine; for example, for the prediction of diabetes risk [8], hypertension [9], drug responses [10], or kidney injury [11].

#### Shapley Additive Explanations

A fundamental feature of studies performed with machine learning techniques is the interpretability of the results. In medicine, this feature is essential for health care professionals to draw conclusions and take decisions based on the results obtained from machine learning algorithms. Doshi-Velez and Kim [12] defined interpretability as the “ability to explain or to present in understandable terms to a human.” This renders interpretability in machine learning a favorable model characteristic.

Recently, the Shapley Additive Explanations (SHAP) framework has been applied to interpret derived machine learning models [13]. SHAP is based on the game theory [14] and helps evaluate feature contributions toward model prediction, identifying the features that most prominently influence the prediction. SHAP values are associated with each feature’s marginal contribution when aggregated to the model. The XGBoost method has an additional advantage when SHAP is used, in that being based on decision trees we can use TreeSHAP, a fast variant of SHAP for tree-based machine learning [15].

### Model Training and Evaluation

In order to obtain a mortality predictive model (“Outcome” feature), a gradient boosting model was trained using previously described data. Input features were “Age,” “Sex,” and each of the laboratory values (mean values) in accordance with the data preprocessing described above. For this, the XGBoost model was developed using the existing implementation for Python.

To initially assess the performance of the XGBoost algorithm in relation to other models in the literature, a comparison was made with 8 representative classifiers in machine learning: decision tree, K-nearest neighbors, linear discriminant analysis, logistic regression, multilayer perceptron, Gaussian naive Bayes, random forest, and support vector machines. For this, the corresponding implementation in the Python Scikit-learn library [16] was used. The metrics analyzed were the area under the receiver operator characteristic curve (AUROC), the area under the precision-recall curve (AUPRC), accuracy, and F-score (F1). To assess a value for these metrics, bootstrap validation was used.

For each classifier, the most relevant model parameters (hyperparameters) were adjusted by selecting the best values after an iterative tuning procedure, and leaving the rest with their default values. Hyperparameter values were identified using hyperopt, a Python library for distributed hyperparameter optimization [17]; the metric and the algorithm used in the optimization were AUROC and the 3-structured Parzen estimator. To estimate the AUROC value, k-fold stratified cross-validation (k=10) was employed. Thus, each tuning cycle involved 10 training-test executions using different nonoverlapping test data (each with 10% of the total records). Through cross-validation, the variance of the estimates can be reduced, and the estimation of the generalization performance was improved [18].

Once the results were analyzed, suitable behavior was confirmed in most of the metrics obtained using XGBoost. To further improve its performance, the final model parameters were adjusted using a more exhaustive tuning procedure. Among the variety of parameters available in XGBoost, the ones considered more relevant were selected for tuning. The 6 selected parameters influence the number of gradient boosted trees and their structure (n_estimators, max_depth, and min_child_weight), and the learning process (learning_rate, subsample, and colsample_bytree).

**Figure 1:**
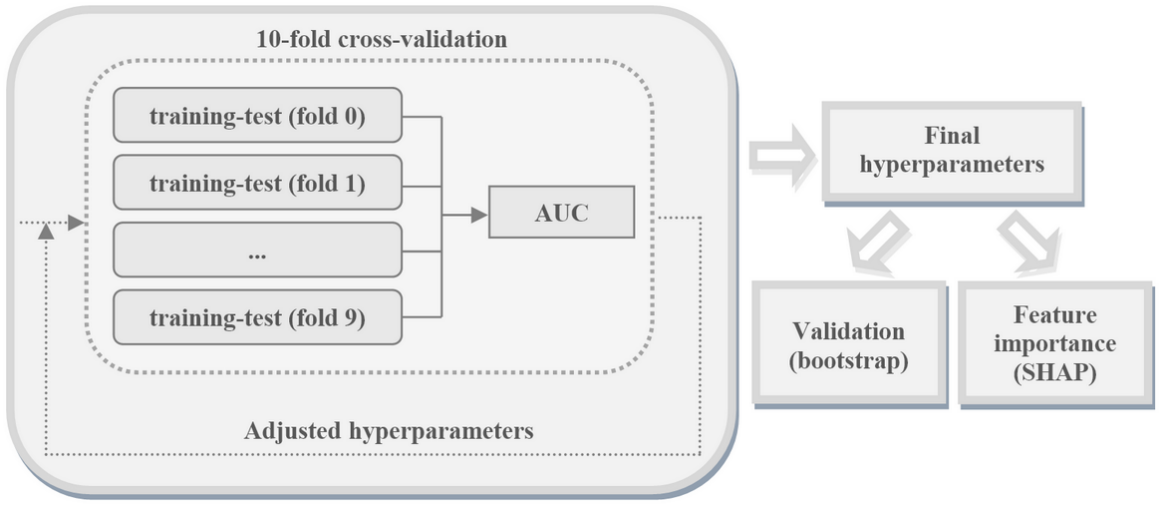
Procedure for obtaining the model parameters, validation, and feature importance. AUC: area under the curve, SHAP: Shapley Additive Explanations.

Following this parameter tuning phase, the final model was assessed through bootstrapping. The performance metrics were as follows: AUROC, AUPRC, accuracy, F1, Youden's index, sensitivity, and specificity. Finally, the relative importance of the features in the model was obtained using SHAP (Figure 1).

## Results

### Study Population and Features

Following the initial data preprocessing phase, the combination of the data in the 2 tables produced a data set composed of 1823 records and 33 features. All the data correspond to patients with a confirmed diagnosis of COVID-19. Tables 1 and 2 show prevalence and clinical laboratory values, respectively. The median age of all patients was 68 (IQR 57-79) years, and 1114 (61.1%) were male. The “Death” outcome had a prevalence of approximately 14% in the resulting subset of patients after data preprocessing.

**Table 1:** Prevalence for the “Age,” “Sex,” and “Outcome” features.

Feature			Patients, n (%)
**Age (years)**			
	0-25		7 (0.4)
	25-50		235 (12.9)
	50-75		942 (51.7)
	75-100		635 (34.8)
	100-125		4 (0.2)
**Sex**			
	Male		1114 (61.1)
	Female		709 (38.9)
**Outcome**			
		Home	1561 (85.6)
		Death	262 (14.4)

**Table 2:** Clinical laboratory values for the features in the data set.

Feature (units)	Median (IQR^a^)	Reference value
Alanine transaminase (U/L)	31.7 (19.2-55.7)	<40
Aspartate transaminase (U/L)	31.8 (22.2-47.3)	<40
Anisocytosis coefficient (%)	13.0 (11.9-14.1)	11.5-14.5
Basophils (%)	0.3 (0.2-0.5)	0-1
Basophil count (10^-3^/µL)	0.02 (0.01-0.03)	0-0.1
C-reactive protein (mg/L)	52.8 (24.1-94.0)	<5
Creatinine (mg/dL)	0.8 (0.7-1.0)	0.6-1.0
D-Dimer (ng/mL)	885 (492-1883)	<500
Eosinophils (%)	0.8 (0.2-1.6)	2-7
Eosinophil count (10^-3^/µL)	0.05 (0.01-0.10)	0.1-0.6
Glucose (mg/dL)	110 (97-132)	70-105
Hematocrit (%)	39.5 (36.5-42.5)	40-54
Hemoglobin (g/dL)	13.3 (12.1-14.3)	13.5-17.5
Lactate dehydrogenase (U/L)	507 (402-654)	120-230
Leukocyte count (10^-3^/µL)	7.0 (5.5-9.2)	4.4-11.3
Lymphocytes (%)	18.4 (12.1-25.5)	20-48
Lymphocyte count (10^-3^/µL)	1.2 (0.9-1.6)	1.2-3.4
Mean corpuscular hemoglobin (pg)	29.7 (28.6-30.8)	28-33
Mean corpuscular hemoglobin concentration (g/dL)	33.5 (32.7-34.2)	33-36
Mean corpuscular volume (fL)	88.4 (85.5-91.5)	80-95
Mean platelet volume (fL)	10.3 (9.7-11.0)	7.4-10.4
Monocytes (%)	8.1 (6.0-10.5)	1-11
Monocyte count (10^-3^/µL)	0.6 (0.4-0.7)	0.1-1
Neutrophils (%)	71.0 (62.5-80.1)	40-75
Neutrophil count (10^-3^/µL)	4.9 (3.6-7.1)	1.5-7.5
Platelet count (10^-3^/µL)	250 (195-317)	150-450
Potassium (mmol/L*)*	4.3 (4.0-4.6)	3.5-5.1
Erythrocyte count (10^-6^/µL)	4.5 (4.1-4.9)	4.1-5.9
Sodium (mmol/L*)*	138 (136-140)	135-145
Urea (mg/dL)	38 (29-54)	5-50

^a^IQR: Q1-Q3 values.

### Model Performance

**Figure 2:**
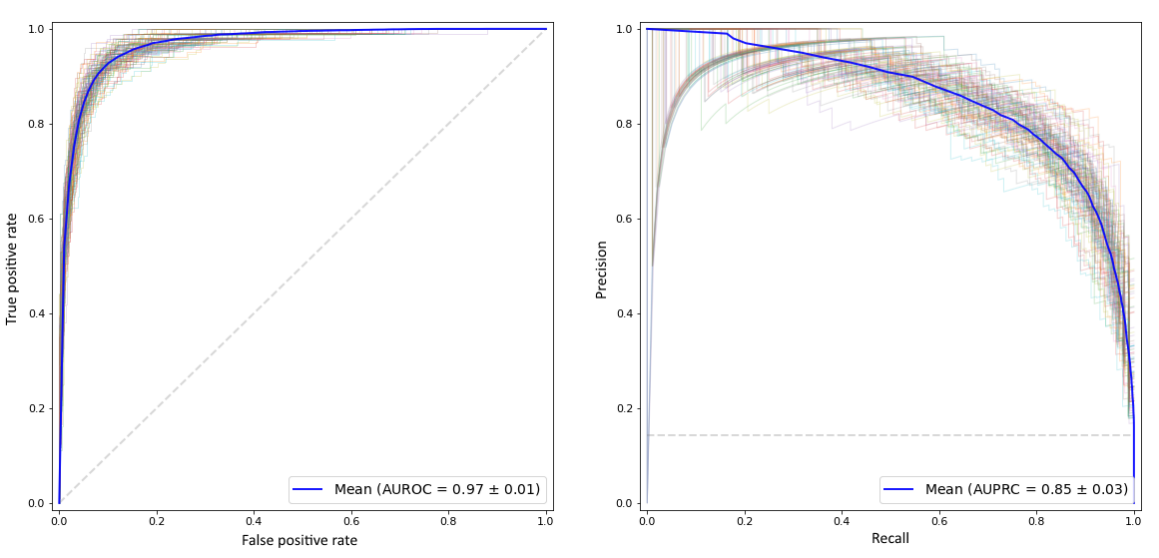
The receiver operator characteristic curve (left) and precision-recall curve (right) in the XGBoost model after bootstrap validation. AUROC: area under the receiver operator characteristic curve, AUPRC: area under the precision-recall curve, XGBoost: extreme gradient boosting.

In the initial evaluation of XGBoost's performance, a comparison with several well-known classifiers was carried out. Table 3 shows the results of this comparison. XGBoost yielded the best results for 3 measures and the second-best results for the F1. These results reaffirm the choice of XGBoost as the predictive method for this study. Figure 2 displays the resulting receiver operator characteristic and precision-recall curves for XGBoost, and Multimedia Appendix 1 shows the corresponding ones for the other methods.

**Table 3:** Comparison of the outcomes of methods after bootstrap validation.

Method	AUROC^a^, mean (95% CI)	AUPRC^b^, mean (95% CI)	Accuracy, mean (95% CI)	F1^c^, mean (95% CI)
Decision tree	0.89 (0.84-0.92)	0.67 (0.58-0.74)	0.89 (0.85-0.92)	0.60 (0.52-0.68)
K-nearest neighbors	0.87 (0.85-0.90)	0.55 (0.46-0.64)	0.88 (0.86-0.90)	0.41 (0.29-0.50)
Linear discriminant analysis	0.96 (0.94-0.97)	0.85 (0.80-0.90)	0.94 (0.92-0.95)	0.75 (0.70-0.82)
Logit	0.96 (0.94-0.98)	0.84 (0.79-0.89)	0.94 (0.92-0.95)	0.76 (0.70-0.82)
Multilayer perceptron	0.95 (0.93-0.97)	0.79 (0.71-0.86)	0.93 (0.91-0.94)	0.73 (0.65-0.79)
Naive Bayes	0.94 (0.91-0.96)	0.74 (0.66-0.82)	0.91 (0.89-0.92)	0.68 (0.62-0.76)
Random forest	0.96 (0.95-0.98)	0.84 (0.76-0.90)	0.93 (0.91-0.95)	0.73 (0.67-0.79)
Support vector machines	0.91 (0.88-0.94)	0.62 (0.53-0.71)	0.87 (0.85-0.88)	0.21 (0.11-0.31)
XGBoost	0.97 (0.96-0.98)	0.85 (0.79-0.91)	0.94 (0.92-0.95)	0.76 (0.71-0.81)

^a^AUROC: area under the receiver operating characteristic curve.

^b^AUPRC: area under the precision-recall curve.

^c^F1: F-score.

In an attempt to improve XGBoost's performance, the final model parameters were adjusted through a more exhaustive tuning procedure using the Python hyperopt library. Setting the number of iterations (max_eval) at 8000 yielded the hyperparameter values presented in Table 4. The remaining hyperparameters retained their default values. The model used 110 decision trees, with a maximum depth of 3.

**Table 4:** Final values of the tuned hyperparameters in the extreme gradient boosting model.

Hyperparameter	Value
Number of gradient-boosted trees	110
Maximum tree depth	3
Minimum sum of instance weight needed in a child	5
Boosting learning rate	0.094
Subsample ratio of the training instances	0.928
Subsample ratio of columns	0.474

With these hyperparameter values, bootstrap validation was again used to obtain model results for the performance metrics, after 300 bootstrap iterations. Through this process, the values obtained were 0.97 (95% CI 0.96- 0.98) for AUROC, 0.86 (95% CI 0.80- 0.91) for AUPRC, 0.94 (95% CI 0.92-0.95) for accuracy, and 0.77 (95% CI 0.72- 0.83) for the F1. We observed a slight improvement owing to more processing in the hyperparameter search process (8000 vs 1000 iterations). Furthermore, the associated sensitivity and specificity values were calculated using the receiver operator characteristic curve values to determine the cut-point that maximizes the Youden index. These calculations yielded a value of 0.85 (95% CI 0.80-0.90) for the Youden index, 0.93 (95% CI 0.89-0.98) for sensitivity, and 0.91 (95% CI 0.86-0.96) for specificity.

### Feature Importance

**Figure 3:**
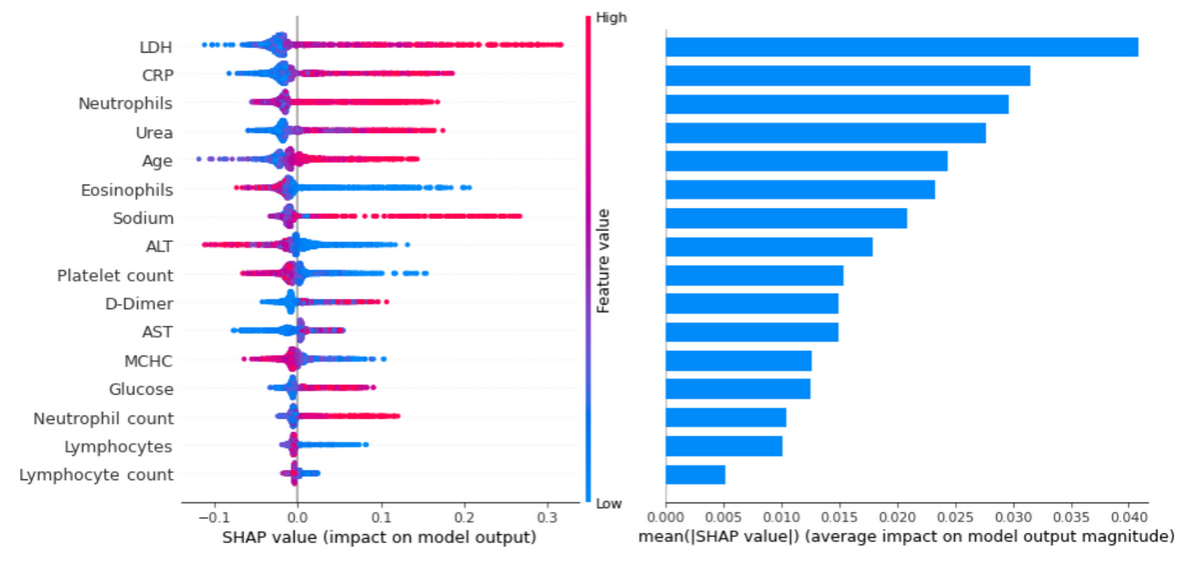
SHAP summary plots for the 16 most important features in accordance with their mean absolute values. Beeswarm plot (left), where each dot corresponds to an individual patient, showing the impact of the feature on the model’s prediction for that patient. The graph on the right shows the average impact on model output. ALT: alanine transaminase, AST: aspartate transaminase, CRP: C-reactive protein, LDH: lactate dehydrogenase, MCHC: mean corpuscular hemoglobin concentration, SHAP: Shapley Additive Explanations.

After applying the tuned XGBoost model to the total data set, the SHAP values associated with this model were calculated. Each feature’s overall performance can be determined on the basis of these SHAP values in accordance with their average impact on model output. Figure 3 shows SHAP summary plots for the 16 most important features. Based on the mean absolute SHAP values, 5 features, including lactate dehydrogenase, C-reactive protein, neutrophil (%), urea, and age, had a greater average impact on model output. Among these, the feature’s highest values (red) are generally associated with a higher SHAP value and, by extension, to a greater likelihood of the “Death” outcome. In other cases, for example eosinophil (%) and alanine aminotransferase, the feature’s lowest values (blue) are associated with a greater risk of the “Death” outcome.

Plots developed using SHAP values are displayed in Multimedia Appendix 1; these highlight the relationship between these features and the mortality risk. Every dot represents an individual patient. Furthermore, Multimedia Appendix 1 contains boxplots that describe value distribution between recovered and dead patients for the same features.

## Discussion

### Principal Findings

COVID-19 mortality is strongly linked to 2 events. There are patients who develop a severe inflammatory syndrome, which results in uncontrolled activation of the immune system and a massive release of proinflammatory cytokines, which translates into an increase in acute-phase reactants such as C-reactive protein, interleukin-6, ferritin, cell destruction markers such as lactate dehydrogenase, and an increase in proinflammatory cells such as neutrophils. This severe inflammatory syndrome has been described as a cause of mortality in most patients with complications arising from a SARS-CoV-2 infection. In such patients, lactate dehydrogenase is associated with an increase in cell destruction, which results in a reduction in lymphocytes, rupture of the lung parenchyma due to inflammation, cell damage, cell remodeling, and lung fibrosis [19,20]. Our study data are concurrent with this trend, with lactate dehydrogenase, C-reactive protein, and neutrophils having the greatest impact on mortality among these patients. Another important complication described in these patients is acute renal failure [21]; our data show that the laboratory parameter that most influences mortality in relation to renal function is urea, a marker of renal function at the prerenal level, which indicates whether renal filtering is effective. Urea levels tend to increase when patients are dehydrated or experience excessive fluid loss [3].

From among clinical laboratory findings, it is essential to establish a biochemical panel of acute-phase reactants that facilitate the identification of patients susceptible to an acute inflammatory syndrome. In this case, we propose lactate dehydrogenase and C-reactive protein as the best candidates according to the data obtained, to which interleukin-6 ferritin should be added, at the very least.

Another complication that results in high mortality in these patients is coagulation disorders. COVID-19 results in a systemic hypercoagulation state, producing pulmonary thromboembolisms, ischemic strokes, and other disorders, and a markedly large number patients experience severe complications. This complication can be assessed on the basis of 2 laboratory parameters: D-Dimer and platelets. As a degradation product of a previously formed clot, the increase in this parameter will thus be proportional to the number of previously formed clots. In the first step of the coagulation process, a reduction in the number of platelets would indicate that clots are being formed. Accordingly, a risk factor would be an increase in D-dimer levels and a reduction in the platelet count [22].

Approximately 30% of patients with COVID-19 complications have hypercoagulation disorders; hence, it is important to be able to predict these complications in order to establish prophylactic anticoagulant treatment as early as possible in patients in whom this blood disorder is identified. Some studies have compared the hypercoagulation status resulting from COVID-19 to that appearing in patients with an antiphospholipid syndrome, who present with the same complications and in whom the treatment is identical [23]. Of note, we have established a strong relationship between coagulation parameters and mortality in the predictive model we developed in this study.

**Figure 4:**
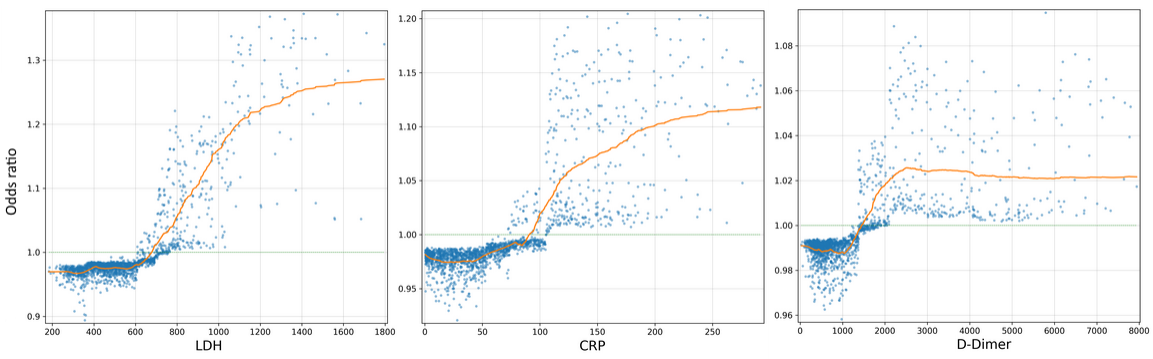
Plots developed using SHAP values, displaying the relationship between laboratory values—including LDH, CRP, and D-Dimer—and mortality risk. Every dot represents an individual patient. Higher values of these features indicate an increase in the mortality risk, and lower ones are associated with a more favorable prognosis. CRP: C-reactive protein, LDH: lactate dehydrogenase, SHAP: Shapley Additive Explanations.

Figure 4 shows the most interesting parameters—from the clinical point of view—and their relation to mortality. The 3 graphs have a common relationship; that is, from a certain value, the curve that relates the value of the variable to mortality increases significantly. At this point, the medical intervention could change the clinical course of patients since, as seen in the graph, very high values in these tests represent a higher mortality risk, while low levels relate to a more favorable prognosis.

**Figure 5:**
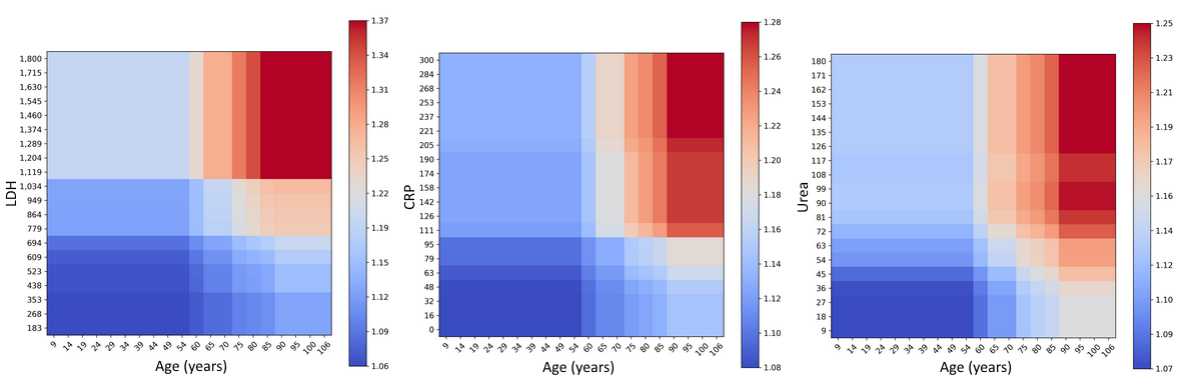
Partial dependence plots representing the model output associated with age and other features (LDH, CRP, and urea). Red zones indicate a greater influence on mortality risk. CRP: C-reactive protein, LDH: lactate dehydrogenase.

**Figure 6:**
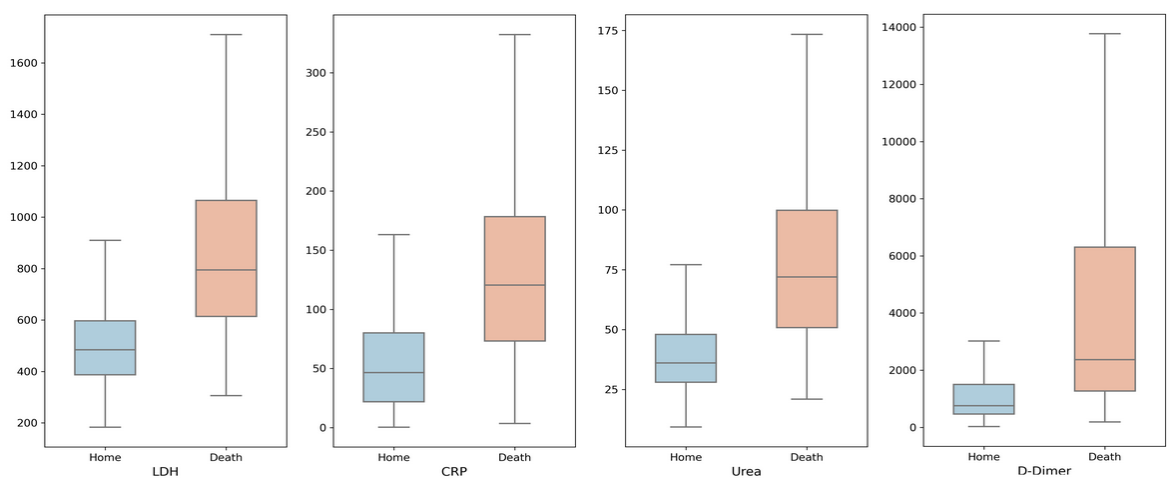
Boxplots describing value distribution between recovered and dead patients on the basis of the laboratory values of LDH, CRP, Urea, and D-Dimer. CRP: C-reactive protein, LDH: lactate dehydrogenase.

At the beginning of the pandemic, one of the main risk factors by which patients were classified was age; as expected, higher morbidity and mortality rates prevail among older individuals. In our predictive model, age ranks in fifth position, which is important, but mortality is still more prominent among those patients who develop a severe inflammatory syndrome. Therefore, if we relate age as an independent variable to the main biochemical markers of severe inflammation, we can estimate patient mortality on the basis of their age and a clinical laboratory value (Figure 5). On the other hand, Figure 6 shows the difference in different clinical laboratory values from among patients who die or are discharged from hospital. We observed a clear difference between different laboratory values depending on each group.

It is not easy to establish strict criteria for mortality in patients with COVID-19, as they are influenced by other unquantified variables and environmental factors. The comorbidities in these patients prior to them contracting COVID-19 are very important when managing these patients and predicting complications [24]. Patients with chronic pathologies such as hypertension or diabetes have a higher number of complications and rate of mortality than those who do not; however, the underlying reason remains unclear. It has been hypothesized that these patients have higher expression levels of angiotensin-converting enzyme 2 receptors through which the virus penetrates the cells to replicate; such patients are candidates for a stronger and more severe disease. Furthermore, Fang et al [25] reported that polymorphisms in the gene that encodes this receptor increases the severity of the disease.

Carrasco-Sánchez et al [26] collected data from approximately 20,000 patients in Spain and reported that mortality can be predicted among those patients who arrive at an emergency department and are then found to have high blood glucose levels, during their hospital stay, provided they are not in a critical condition. Blood glucose is thus one of the most prominent predictors of patient mortality, which is concurrent with our hypothesis. Therefore, glycemic control among patients before and during their hospital stay is essential to increase their survival.

### Limitations

Clinically, this study has a series of limitations. First, this study has a small patient cohort; previous similar studies have included a markedly larger patient cohort [21,26]. Second, we did not record the comorbidities of these patients; therefore, we cannot assess their role in relation to other variables and their potential to predict a patient's mortality. Finally, it is very important to perform a battery of laboratory tests, which facilitates the evaluation of an inflammatory syndrome with more parameters, such as interleukin-6 and ferritin.

### Conclusions

This study aimed to develop a model to predict the mortality of patients with COVID-19, which can assess mortality from laboratory values with a high degree of accuracy. The use of machine learning techniques, in this case the XGBoost predictive method, has yielded excellent results for several performance metrics. The analysis of the resulting model enables us to identify a set of features with a markedly high prediction potential, which can be useful for improving care decisions and increasing patient survival.

## Appendix

Multimedia Appendix 1 Supplementary figures.

## Abbreviations

AUPRC: area under the precision-recall curve
AUROC: area under the receiving operator characteristic curve
SHAP: Shapley Additive Explanations
XGBoost: Extreme Gradient Boosting
