# Supplementary material for: Machine Learning Applied to Clinical Laboratory Data in Spain for COVID-19 Outcome Prediction: Model Development and Validation
Source: J Med Internet Res. 2021 Apr 14;23(4):e26211. doi: 10.2196/26211 (PMC8048712; doi:10.2196/26211)

# Supplementary Figures

Figure S1: Results for receiver operator characteristic curve (left) and precision-recall curve (right) after bootstrap validation, for the methods DT (decision tree) and KNN (K-nearest neighbors).

| 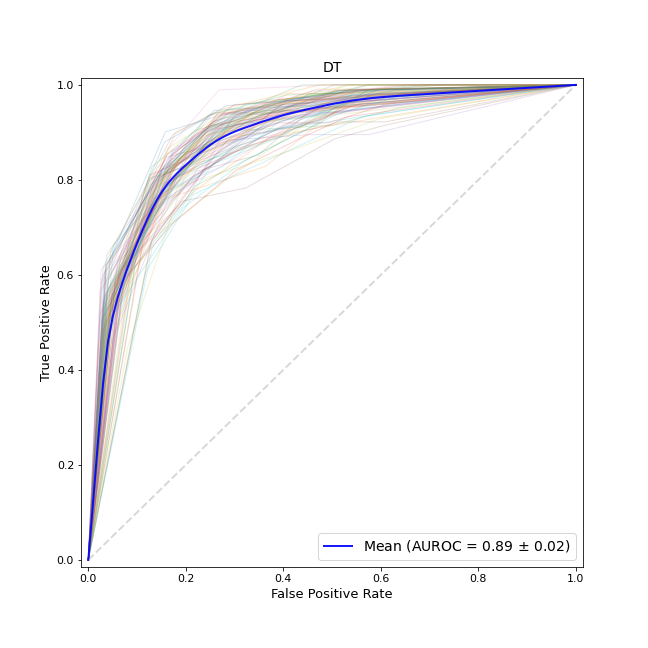 | 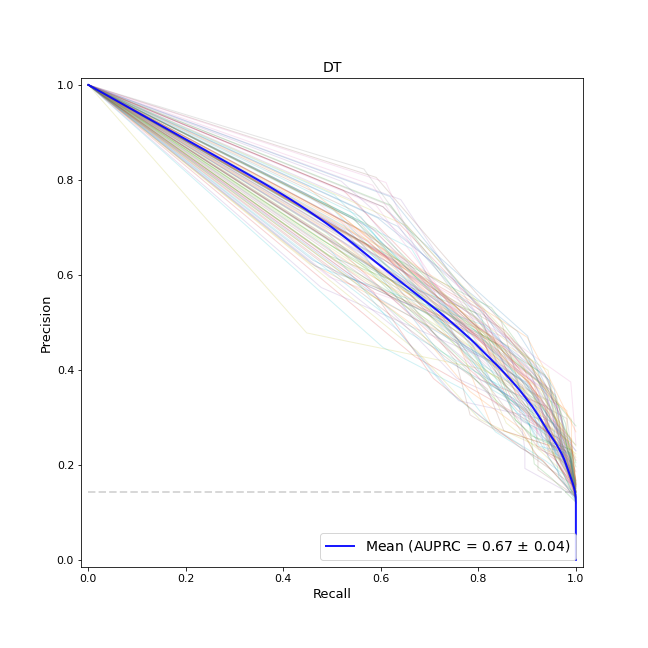 |
| --- | --- |
| 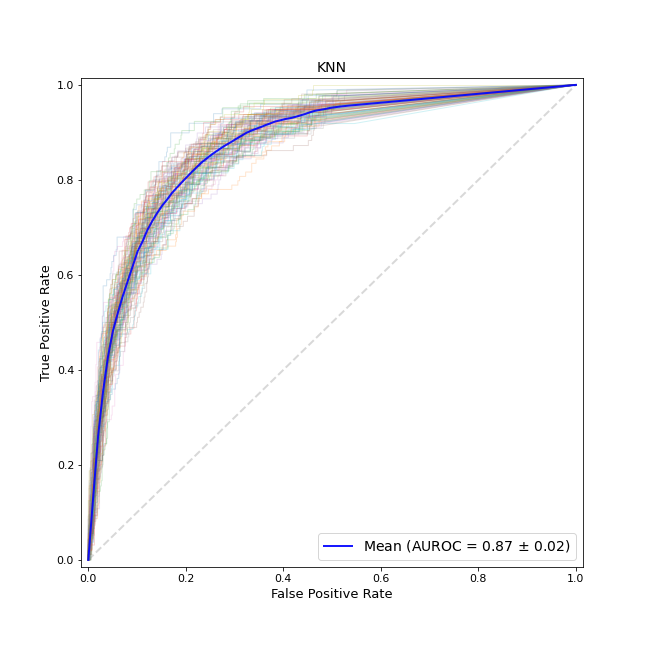 | 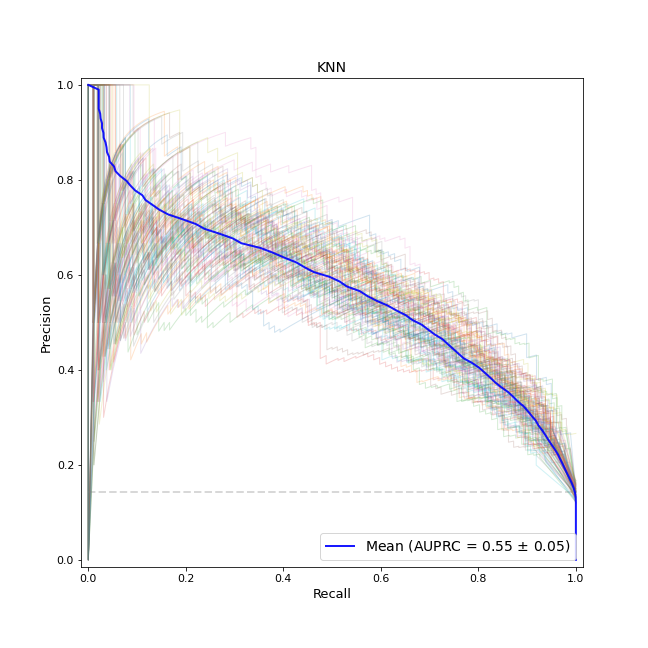 |

Figure S2: Results for receiver operator characteristic curve (left) and precision-recall curve (right) after bootstrap validation, for the methods LDA (linear discriminant analysis) and Logit (logistic regression).

| 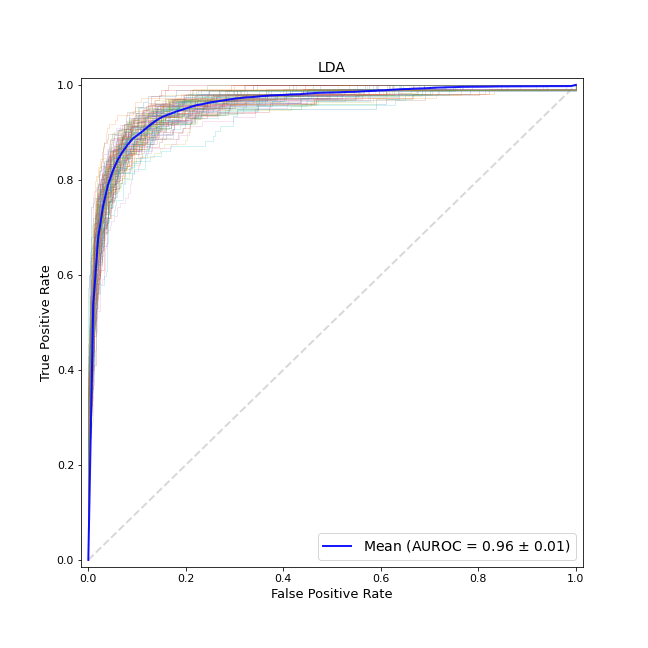 | 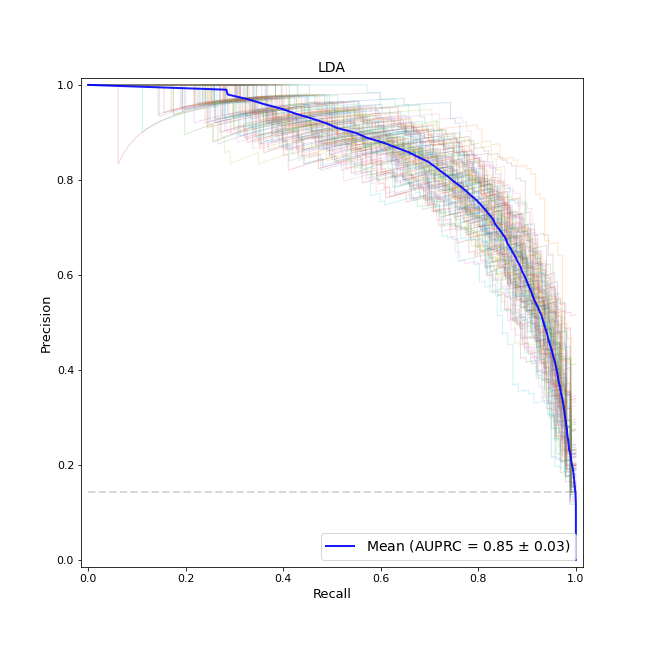 |
| --- | --- |
| 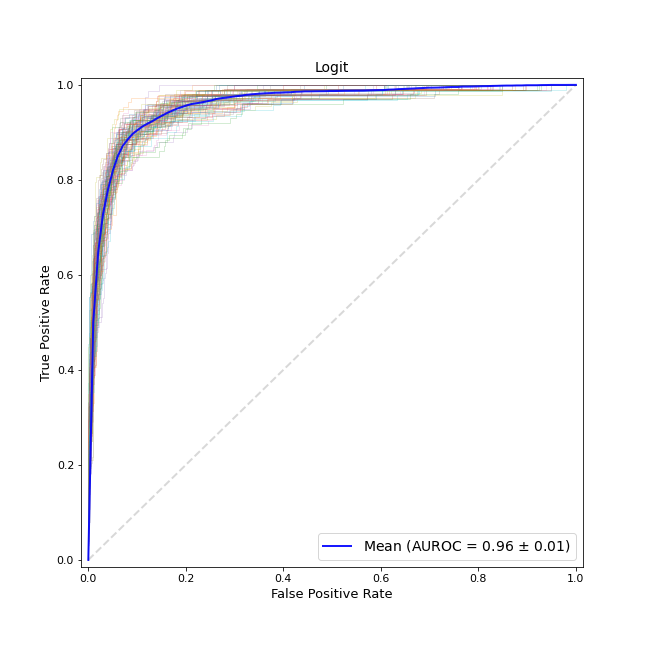 | 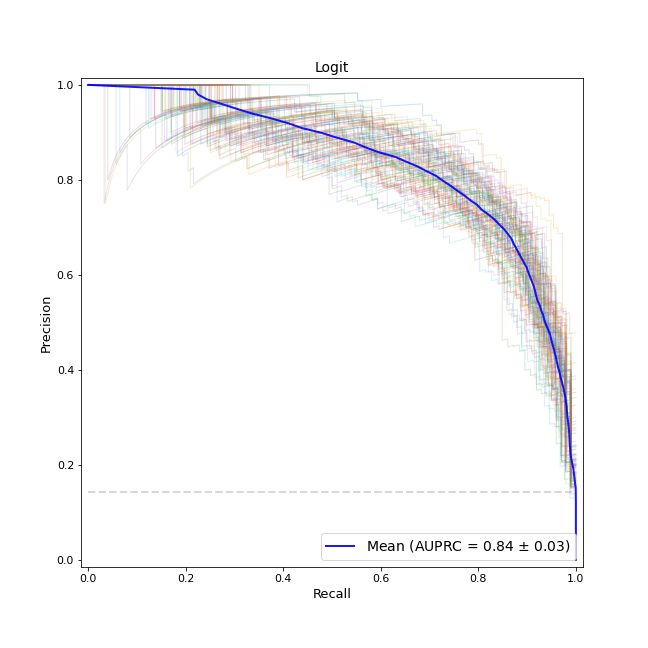 |

Figure S3: Results for receiver operator characteristic curve (left) and precision-recall curve (right) after bootstrap validation, for the methods MLP (multi-layer perceptron) and NB (naive Bayes).

| 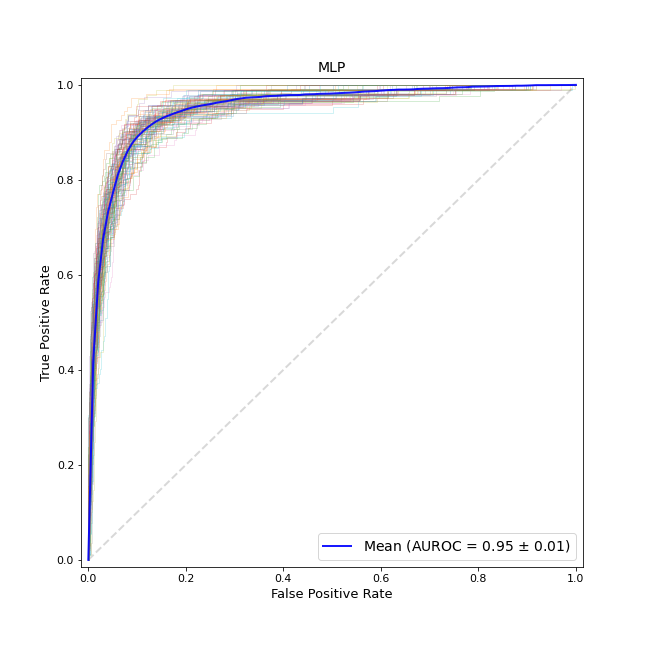 | 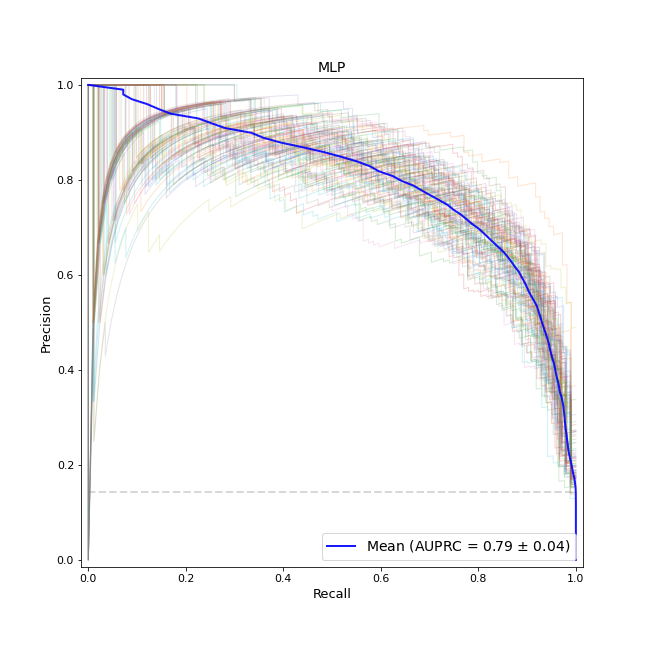 |
| --- | --- |
| 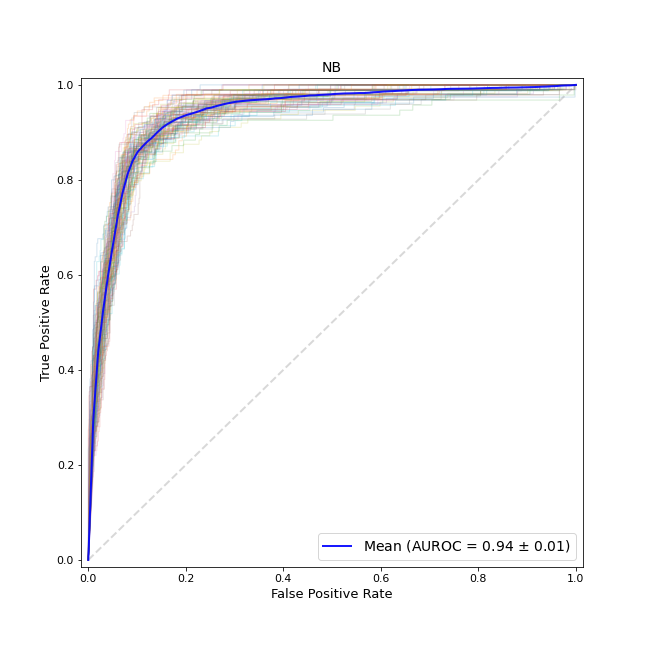 | 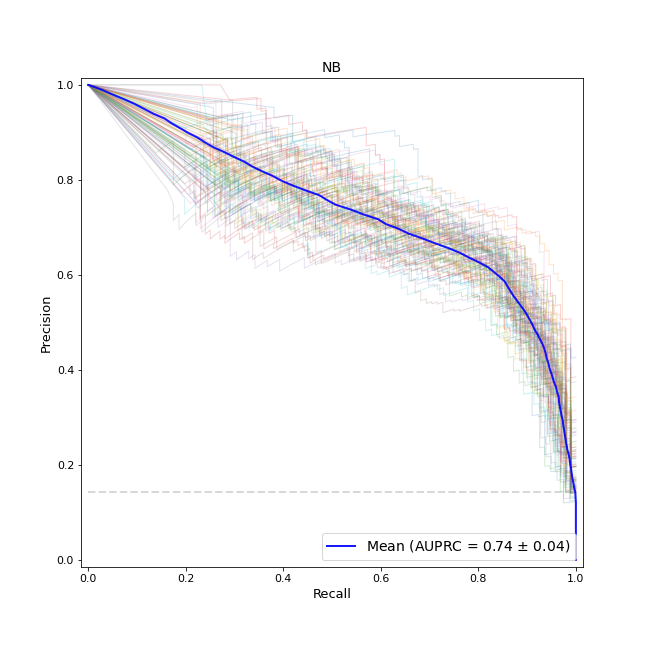 |

Figure S4: Results for receiver operator characteristic curve (left) and precision-recall curve (right) after bootstrap validation, for the methods RF (random forest) and SVM (support vector machines).

| 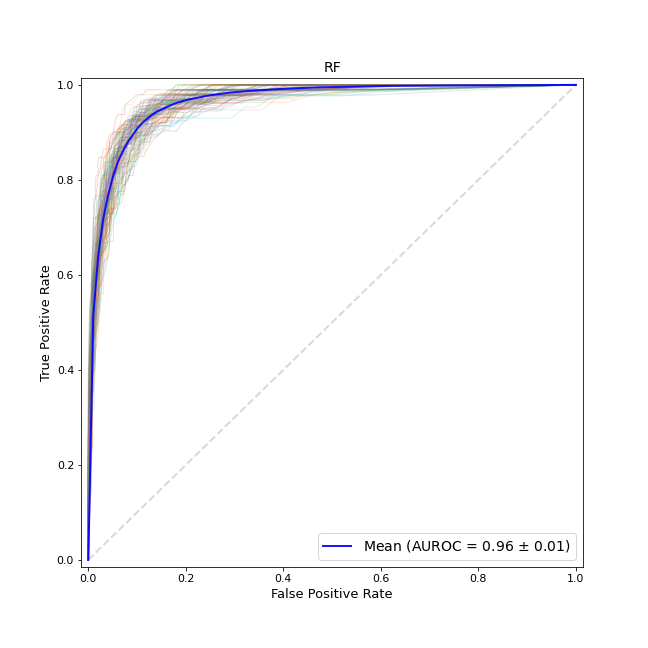 | 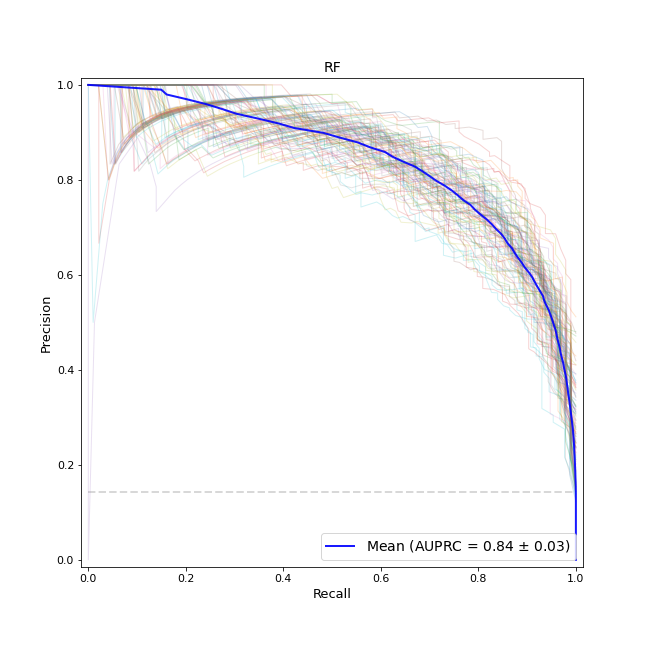 |
| --- | --- |
| 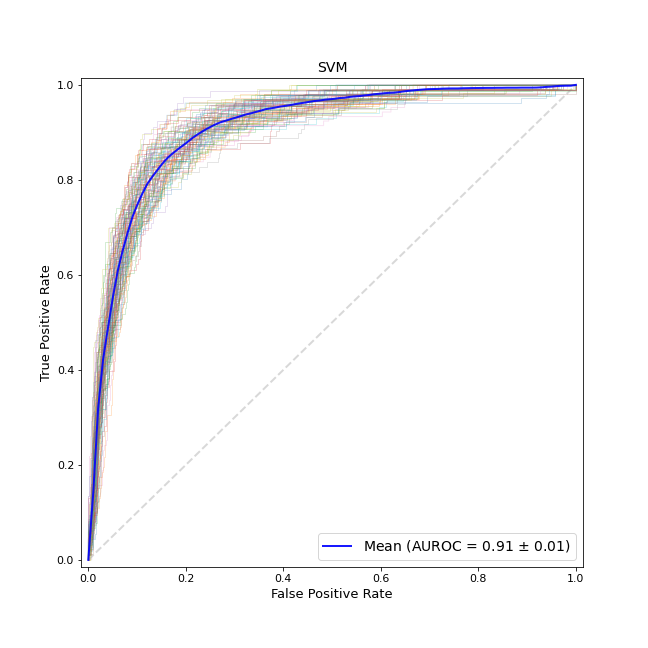 | 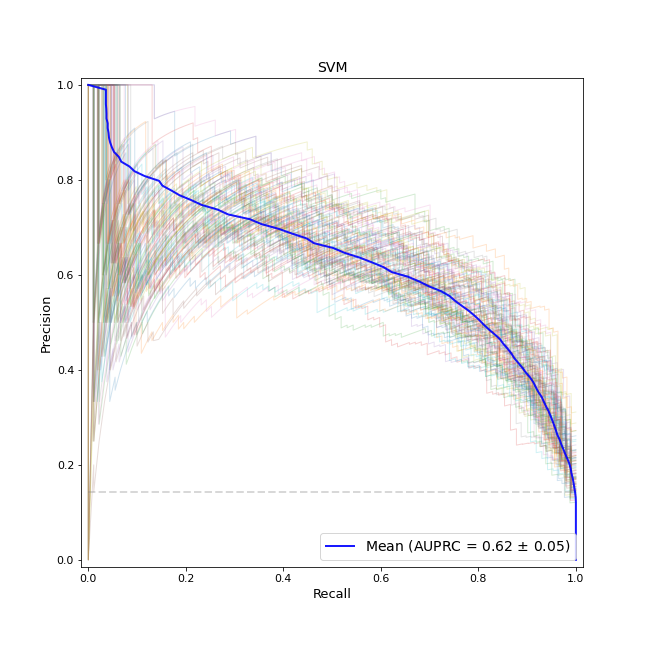 |

Figure S5: Plots developed using SHAP values, displaying the relationship between the features (LDH, CRP, Neutrophils, and Urea) and mortality risk.


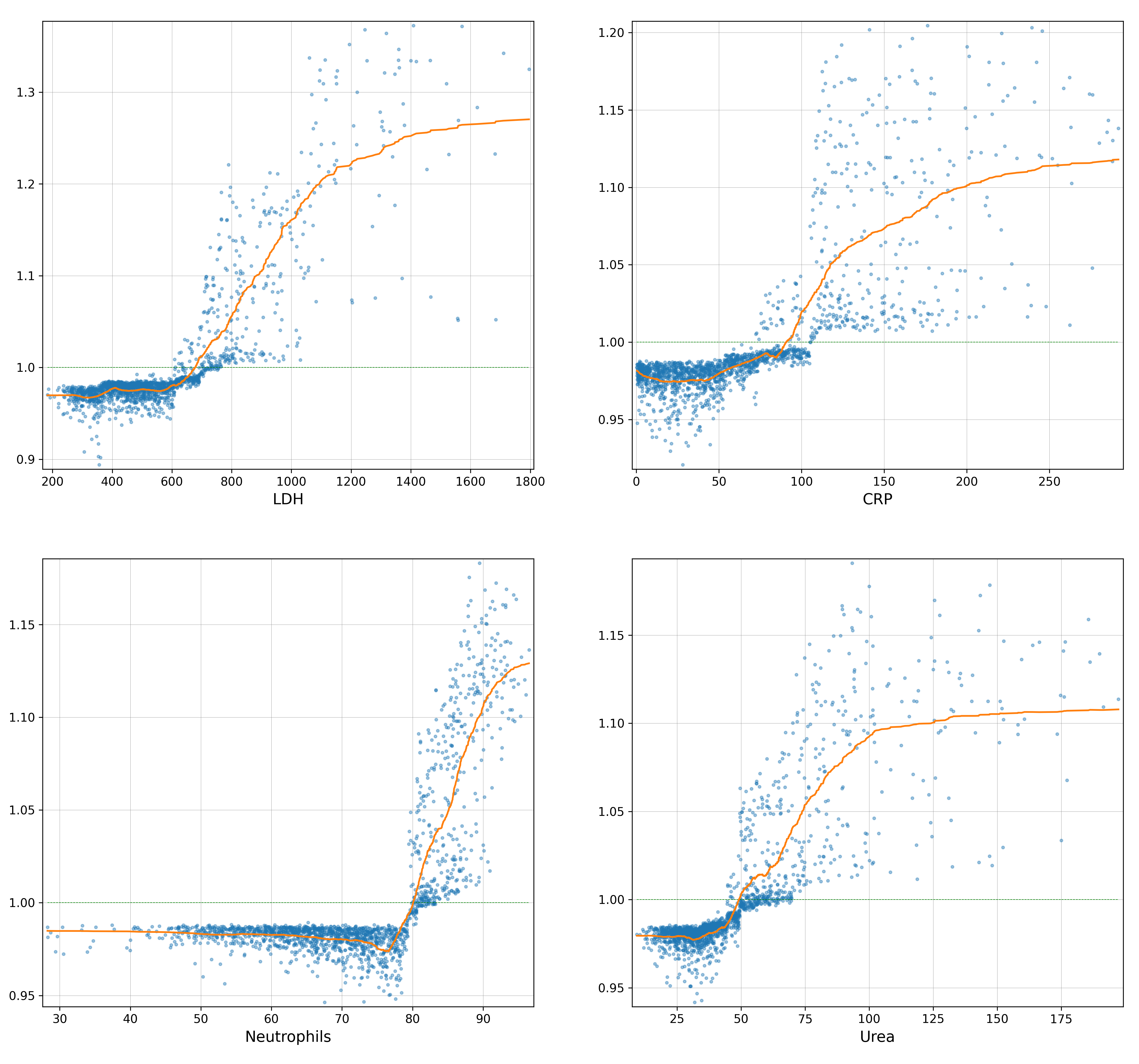


Figure S6: Plots developed using SHAP values, displaying the relationship between the features (Age, Eosinophils, Sodium, and ALT) and mortality risk.


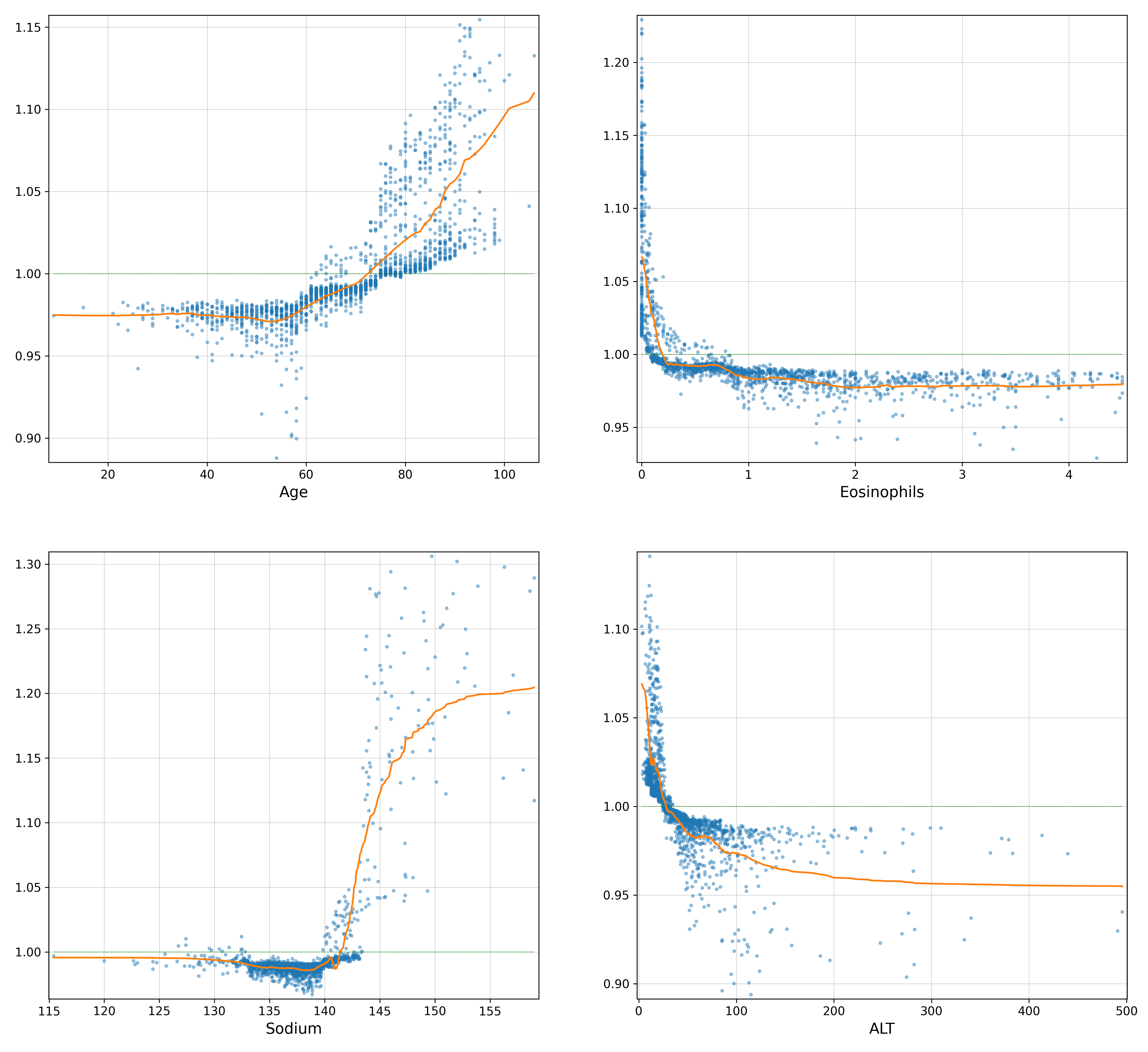


Figure S7: Plots developed using SHAP values, displaying the relationship between the features (Platelets count, D-Dimer, AST, and MCHC) and mortality risk.


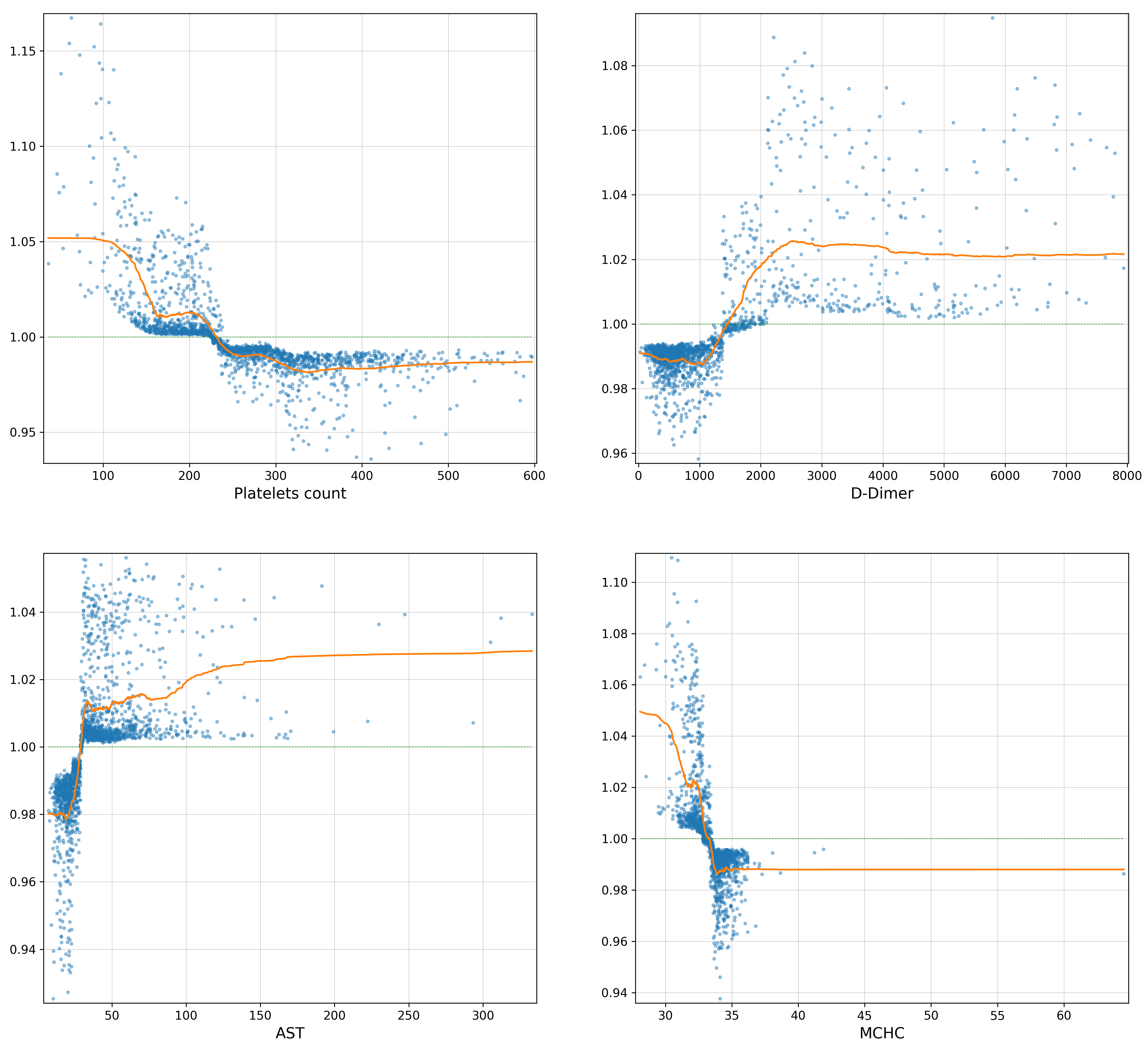


Figure S8: Plots developed using SHAP values, displaying the relationship between the features (Glucose, Neutrophils count, Lymphocytes, and Lymphocytes count) and mortality risk.


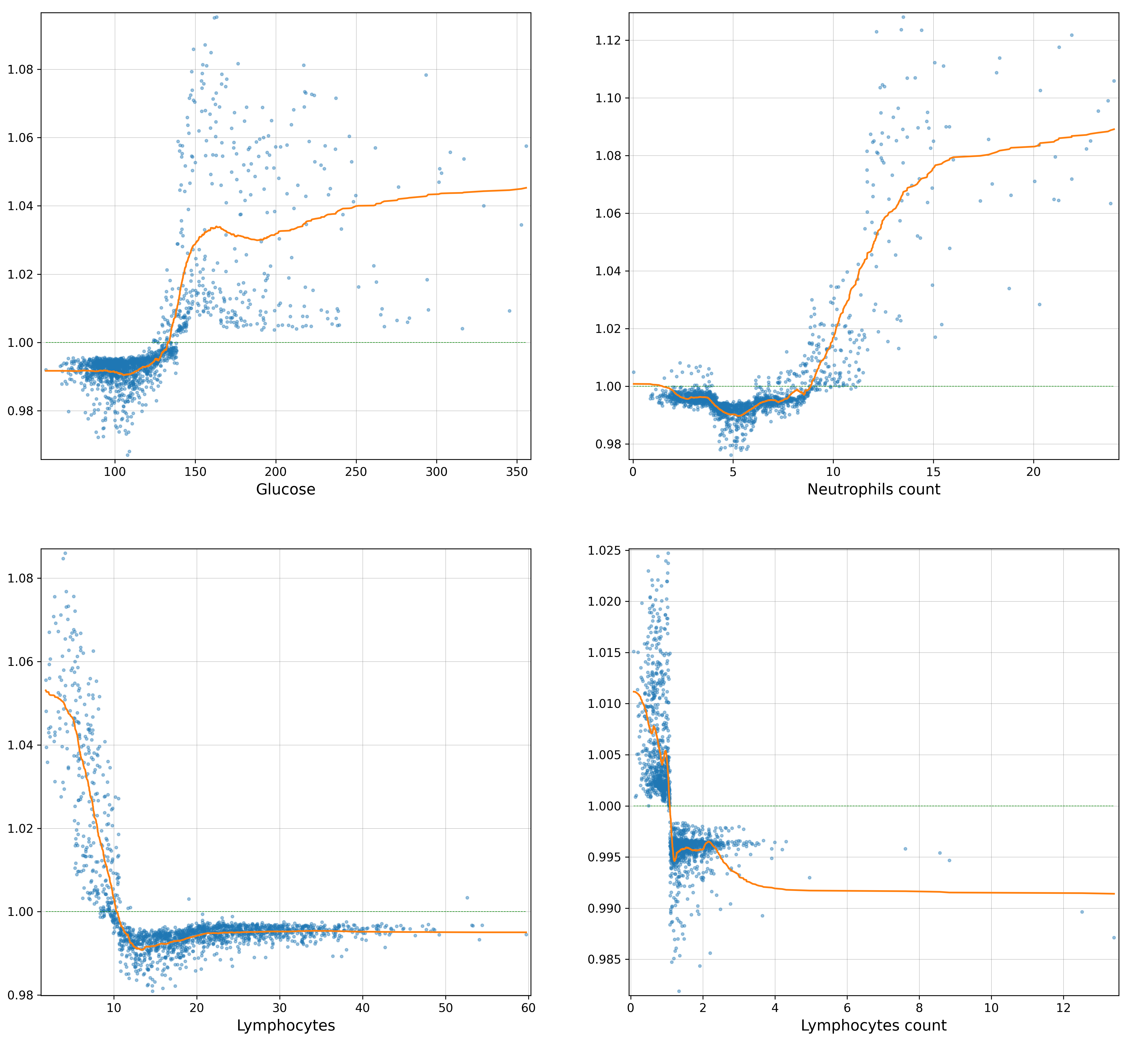


Figure S9: Box-plots describing value distribution between recovered and dead patients, for LDH, CRP, Neutrophils, Urea, Age, Eosinophils, Sodium, and ALT).


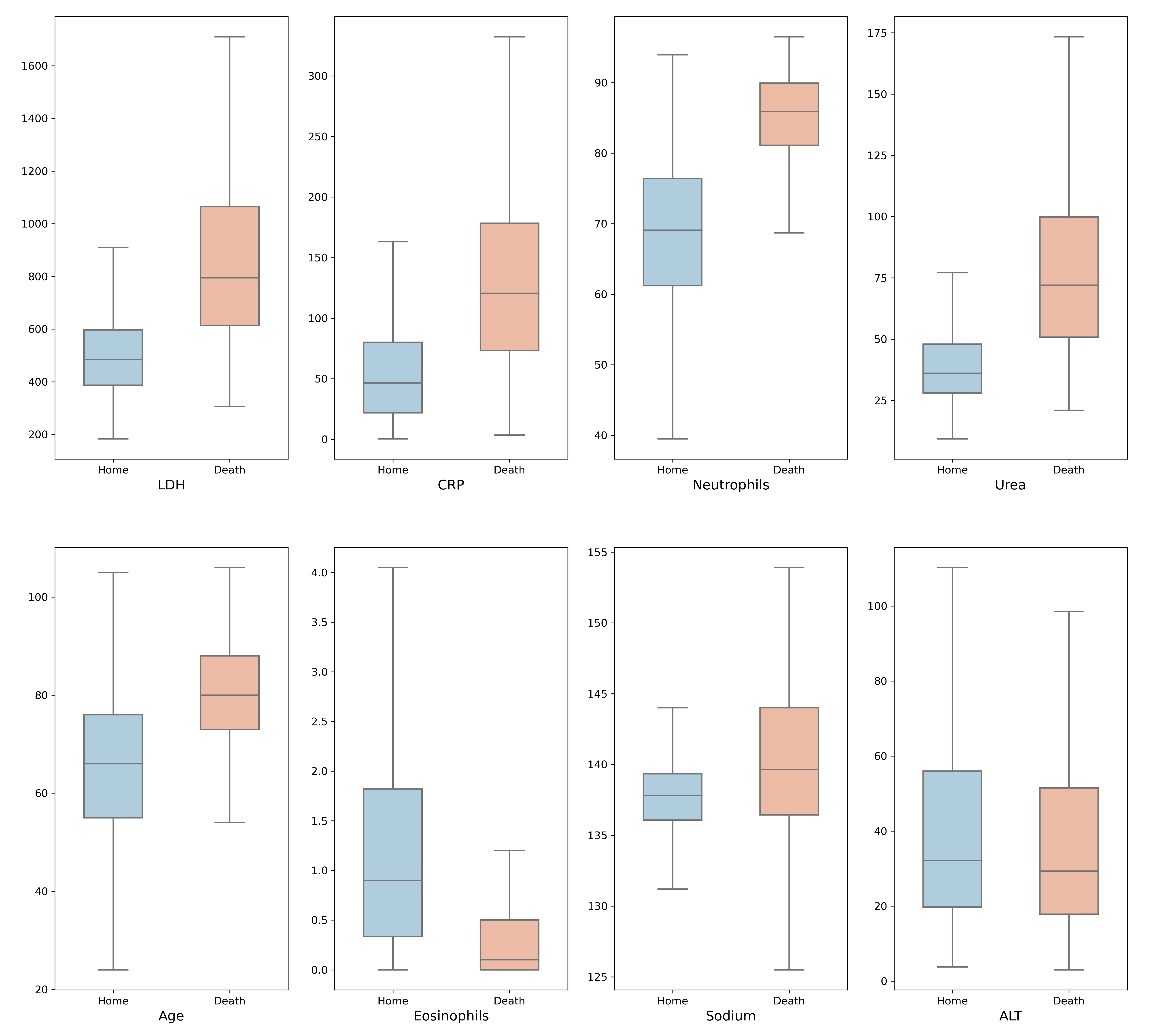


Figure S10: Box-plots describing value distribution between recovered and dead patients, for Platelets count, D-Dimer, AST, MCHC, Glucose, Neutrophils count, Lymphocytes, and Lymphocytes count.


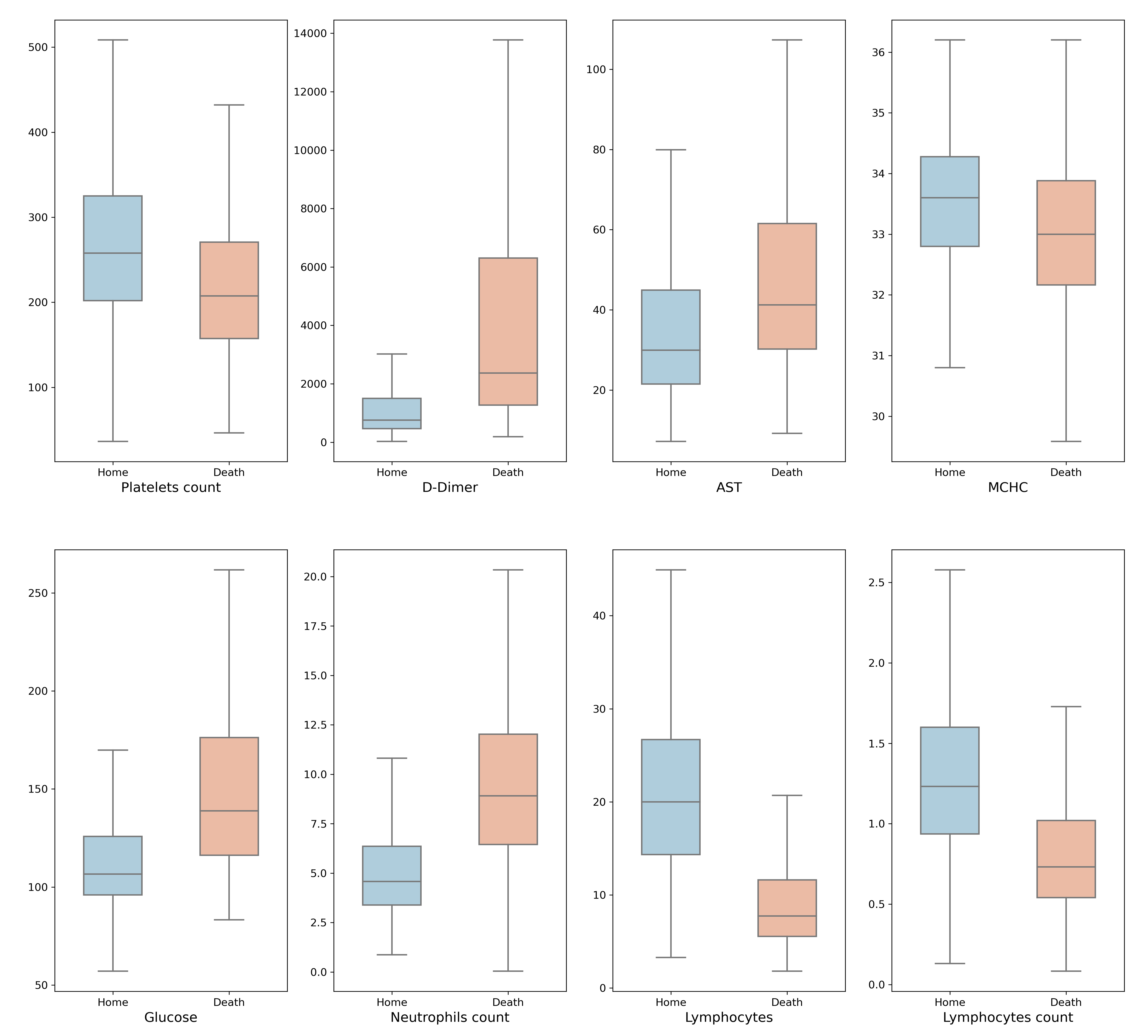

Supplement: Multimedia Appendix 1 [file jmir_v23i4e26211_app1.docx]
